# From the archives: Stress signaling—U-box proteins in the cold stress response, sensor activation in response to salt stress, and early work on salicylic acid signaling

**DOI:** 10.1093/plcell/koae186

**Published:** 2024-06-26

**Authors:** Rory Osborne

**Affiliations:** Assistant Features Editor, The Plant Cell, American Society of Plant Biologists; School of Biosciences, University of Birmingham, Birmingham B15 2TT, UK

## 2023: PUB25 and PUB26 regulate adaptive responses to cold stress

Plants must respond to short-term stresses, but a regulated and proportionate response is essential for long-term survival. A tightrope walker crossing a high wire must balance every step with precision—overreacting to a gust of wind would be as dangerous as not reacting at all. One example of this negative feedback is the expression of the master transcriptional regulators *C-REPEAT BINDING FACTOR1/2* (*CBF1/2*) during the cold response. Upon low temperature perception, *CBF1/2* are massively induced during the first hour but rapidly return to steady state after only 6 h.

Wang et al. 2023 sought to understand how this critical facet of the cold response is regulated in *Arabidopsis thaliana* by exploring the activity of 2 E3 ligases, *PLANT U-BOX 25* and *PLANT U-BOX 26* (PUB25 and PUB26; Wang et al. 2023). In their recent article, the authors showed that *INDUCER OF CBF EXPRESSION1* (*ICE1*)—a gene well known to positively regulate the cold response by inducing *CBF1*—is a direct target of these E3 ligases. Using a series of biochemical and molecular techniques, the authors demonstrate that PUB25 and PUB26 differentially regulate ICE1 through the ligation of both K48- and K63-linked poly-ubiquitin chains. While K48 chains typically promote 26S proteasome-dependent proteolysis, K63 is more commonly associated with post-translational regulation of protein function. Given that the differential ligation of these chains was time dependent, with K63 preceding K48 over 6 h, the authors conclude that PUB25 and PUB26 initially activate ICE1 during early cold responses but then later destabilize it to attenuate its activity.

This regulatory module was further defined when PUB25/26 were shown to also target MYB15 for degradation during the first hour of cold treatment. MYB15 negatively regulates the cold response by transcriptionally inactivating *CBF1*: its targeted destruction during early cold treatment thus supported enhanced freezing tolerance. Finally, the authors show that stabilized K63 linked ICE1 directly inhibits MYB15 binding to the *CBF3* promoter, thus generating a full picture of the mechanistic basis for dynamic *CBF* expression during the cold response.

## 2019: Mechanistic insight into salt tolerance of rice

Low temperatures are not the only abiotic factors that impact plant growth; flooding, drought, and elevated soil salinity all require adaptive responses to promote survival. Zhao et al. 2019 investigated the early signaling events regulating salt stress responses in rice, for which the picture is not as detailed as in Arabidopsis. Building on their previous work, which characterized the *Oryza sativa* receptor-like kinase (RLK) SALT INTOLERANCE 1 (*OsSIT1*) as a sensor and positive regulator of salt tolerance (Li et al. 2014), the authors demonstrate that the activity of this RLK is regulated by the B'κ subunit of the Protein Phosphotase 2A complex. By generating a suite of CRISPR knockouts and phosphatase-null overexpressors, the authors show that the B'κ regulatory subunit of PP2a is also a positive regulator of salt tolerance in rice. Using a series of in vitro phosphorylation assays and mass spectrometry, they next define the catalytic loop of OsSIT1, responsible for its kinase activity, and identify the residues subject to dephosphorylation by PP2A-B'κ. Interestingly, B'κ and OsSIT1 mutually regulate each other's phospho-status under salt stress conditions. Under nonstress conditions, low levels of B'κ dephosphorylate *OsSIT1* to block its downstream activity when not required. Conversely, under high salinity, B'κ is phosphorylated by *OsSIT1*, which stabilizes it at the protein level. This generates a large pool of activated B'κ, which not only dampens OsSIT1 activity through negative feedback but also promotes salt tolerance by presumably dephosphorylating as-yet-unknown downstream effectors of the salinity response.

## 1999: Identification of ACD6 as a positive regulator of salicylic acid signaling

At the heart of plant stress response are the phytohormones, which support adaptation to different biotic and abiotic factors. Early work by Rate et al. (1999) on salicylic acid signaling revealed a novel gene, *ACCELERATED CELL DEATH 6* (ACD6), as a regulator of plant immunity. A gain-of-function mutant allele of *ACD6* was identified, which exhibited hallmark traits associated with autoimmunity, including overexpression of *PATHOGENESIS RESPONSE1* (*PR1*), dwarfism, and enhanced resistance to the hemibiotroph *Pseudomonas syringae pv Tomato DC3000*. While salicylic acid dependent, *ACD6* function was shown to be partially independent of the master regulator *NONEXPRESSOR OF PR1* (*NPR1*)—a remarkable discovery at the time, given that the then-recently discovered *NPR1* was a major focus for many plant pathologists.

Work has since revealed that *ACD6* encodes a trans-membrane Ca^2+^ channel (Chen et al. 2023) that associates with the Pattern Recognition Receptor FLS2 (Zhang et al. 2014) in response to SA during pattern-triggered immunity. This leap in our understanding of *ACD6* really emphasizes how technological advancement allows us to ask biological questions at higher and higher resolution (see Fig.).

**Figure.**
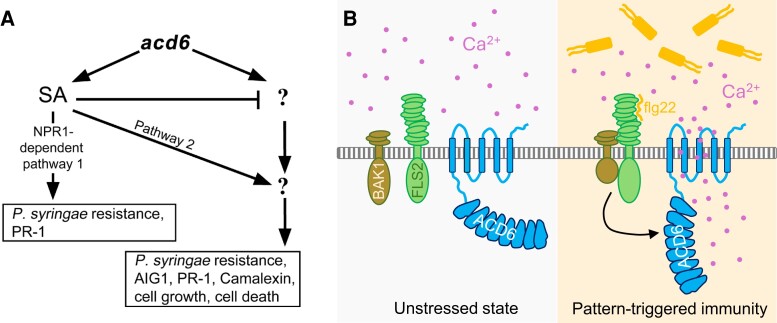
Twenty-five years of increasing resolution: from the original identification and proposed activity of the gain-of-function acd6 mutant (**A**), to the current working model of ACD6 as a Ca2+ ion channel induced during pattern-triggered immunity (**B**). Adapted with permission from Rate et al. (1999), Figure 9, copyright American Society of Plant Biologists (A); and Chen et al. (2023), graphical abstract, copyright Elsevier (B).

## Dive Curated Terms

The following phenotypic, genotypic, and functional terms are of significance to the work described in this paper:

ACD6 Gramene: AT4G14400
ACD6 Araport: AT4G14400

PUB25 Gramene: AT3G19380
PUB25 Araport: AT3G19380

MYB15 Gramene: AT3G23250
MYB15 Araport: AT3G23250

PUB26 Gramene: AT1G49780
PUB26 Araport: AT1G49780

FLS2 Gramene: AT5G46330
FLS2 Araport: AT5G46330

NPR1 Gramene: AT1G64280
NPR1 Araport: AT1G64280

CBF1 Gramene: AT4G25490
CBF1 Araport: AT4G25490
